# The Clinical, Radiologic, and Prognostic Differences Between Pediatric and Adult Patients With Myelin Oligodendrocyte Glycoprotein Antibody-Associated Encephalomyelitis

**DOI:** 10.3389/fneur.2021.679430

**Published:** 2021-05-20

**Authors:** Jie Xu, Lingjuan Liu, Jie Xiong, Lu Zhang, Peng Huang, Li Tang, Yangyang Xiao, Xingfang Li, Jian Li, Yingying Luo, Huiling Li, Dingan Mao, Liqun Liu

**Affiliations:** ^1^Department of Pediatrics, The Second Xiangya Hospital, Central South University, Changsha, China; ^2^Children's Brain Development and Brain Injury Research Office, The Second Xiangya Hospital, Central South University, Changsha, China; ^3^Department of Neurology, The Second Xiangya Hospital, Central South University, Changsha, China; ^4^Department of Ophthalmology, The Second Xiangya Hospital, Central South University, Changsha, China

**Keywords:** myelin oligodendrocyte glycoprotein antibody-associated encephalomyelitis, demyelinating disease, optic neuritis, pediatrics, adults

## Abstract

**Purpose:** To evaluate the clinical differences between pediatric and adult patients with myelin oligodendrocyte glycoprotein antibody-associated encephalomyelitis (MOG-EM).

**Methods:** We retrospectively reviewed the clinical features of pediatric and adult patients with MOG-EM in our center between November 2015 and October 2020.

**Results:** Twenty-eight pediatric patients and 25 adults were admitted to our study. Bilateral optic neuritis (BON) was the most common initial phenotype in the pediatric group but less common in the adult group (28.57 vs. 0%, *p* = 0.0119). Almost half of the adult patients presented with neuromyelitis optica spectrum disease (NMOSD), which was less prevalent among the pediatrics (48 vs. 21.43%, *p* = 0.0414). Visual impairment was the most common symptom in both groups during the initial attack (pediatric group, 39.29%; adult group, 64%) and throughout the full course (pediatric group, 57.14%; adult group, 72%). More pediatric patients suffered from fever than adult patients at onset (pediatric group, 28.57%; adult group, 4%; *p* = 0.0442) and throughout the full course (pediatric group, 39.29%; adult group, 12%; *p* = 0.0245). Multiple patchy lesions in subcortical white matter (pediatric group, 40.74%; adult group, 45%), periventricular (pediatric group, 25.93%; adult group, 35%), infratentorial (pediatric group, 18.52%; adult group, 30%) and deep gray matter (pediatric group, 25.93%; adult group, 20%) were frequent in all cases, no significant difference was found between the two groups, while bilateral optic nerve involvement was more frequent in pediatric group (61.54 vs. 14.29%, *p* = 0.0042) and unilateral optic nerve involvement was higher in adult group (64.29 vs. 15.38%, *p* = 0.0052). At the last follow-up, adult patients had a higher average EDSS score (median 1.0, range 0–3) than pediatrics (median 0.0, range 0–3), though not significant (*p* = 0.0752). Patients aged 0–9 years (61.54%) and 10–18 years (70%), and patients presenting with encephalitis/meningoencephalitis (100%) and ADEM (75%) were more likely to recover fully.

**Conclusions:** Visual impairment was the dominant symptom in both pediatric and adult patients, while fever was more frequent in pediatric patients. Data suggested that BON and bilateral optic nerve involvement were more common in pediatric cases whereas NMOSD and unilateral optic nerve involvement were more prevalent in adults. The younger patients and patients presenting with encephalitis/meningoencephalitis and ADEM tended to recover better.

## Introduction

Myelin oligodendrocyte glycoprotein (MOG) is a glycoprotein expressed on the outer surface of myelin sheaths in the central nervous system (CNS) (1). Although MOG is a minor component of myelin, it is thought to be a cellular adhesive molecule that maintains the integrity of myelin sheaths and acts as a regulator that mediates the complement cascade (2). Immunoglobulin G (IgG) against MOG is considered to be a potential autoantibody that can induce demyelination in the CNS, and this was supported by the discovery of MOG-IgG in patients with multiple sclerosis (MS) and reports on the clinical relevance of antibodies against myelin oligodendrocyte glycoprotein in different types of MS (3). Recently, MOG-IgG was identified in patients with aquaporin-4 (AQP4) negative neuromyelitis optica spectrum disease (NMOSD) using a cell-based assay (CBA) (4). Besides MS and NMOSD, MOG-IgG is considered to be related to other idiopathic inflammatory demyelinating diseases (IIDDs), including acute disseminated encephalomyelitis (ADEM), optic neuritis (ON), transverse myelitis (TM), and clinically isolated syndrome (CIS) (5–7). Several researchers have suggested that MOG antibody-related demyelinating disease is an independent disease with unique clinical manifestations, radiologic presentations, treatment response, and prognosis, and it differs from other IIDDs. It is currently defined as MOG antibody-associated encephalomyelitis (MOG-EM) (8, 9).

Several studies have demonstrated that pediatric- and adult-onset cases of MOG-EM have different clinical characteristics (10–12); however, these findings vary with studies, countries, and populations, and most studies are case series or they involve small samples. In this study, we aimed to evaluate the different clinical manifestations, radiologic presentations, and prognoses of pediatric and adult patients with MOG-EM in a relatively large cohort in China.

## Materials and Methods

### Patients

We retrospectively collected data from 28 patients aged ≤18 years and 25 patients aged >18years who met the 2018 diagnostic criteria for MOG-EM (9) and were admitted to the Second Xiangya Hospital of the Central South University between November 2015 and October 2020. Age from 0 to 14 years was defined as pediatric group, and age over 18 years was defined as adult group. Two patients with age 16 and 17 years were also enrolled into pediatric group since them were admitted, treated and followed up in pediatric department. These patients were enrolled from three departments of the Second Xiangya Hospital, including the Pediatric Department, Neurology Department, and Ophthalmology Department. This study was approved by the ethics committee of the hospital.

### Definitions

The criteria for the IIDDs including NMOSD, ADEM and MS were in accordance with the diagnostic criteria proposed by the International Multiple Sclerosis Study Group (IPMSSG) in 2013 (13). The “BON” refers to the clinical disease of bilateral optic neuritis, and “bilateral optic nerve involvement” refers to the optic nerve MRI findings at acute stage of disease by using T2- and T1-weighted imaging. Relapse was defined as new CNS demyelination symptoms or signs that developed at least 30 days after the first episode and lasted for over 24 h. The outcome was evaluated using the Expanded Disability Status Scale (EDSS) score, which was assessed during the peak of the initial attack and at the end of follow-up. The EDSS score was only evaluated in patients with follow-up durations of >3 months. Recovery was graded as follows: full, EDSS = 0; moderate, 0 < EDSS < 3; poor, EDSS≥3.

### Laboratory Examinations

All patients were tested for serum anti-MOG-IgG and anti-AQP4-IgG by the Simsere Laboratory and Kindstar Global Laboratory using a MOG-IgG1 specific cell-based assay (CBA) with HEK293 cells transfected with the full-length human MOG and AQP4 antigen (9). The CSF white blood cell (WBC) count, total protein, serum autoantibodies, and other biochemical examinations were performed by the Department of Clinical Laboratory of the Second Xiangya Hospital.

### Magnetic Resonance Scanning

Brain, optic nerve, and spinal cord magnetic resonance scanning were performed using T2- and T1-weighted imaging with and without gadolinium enhancement at acute stage and before treatment. All images were analyzed by two researchers who were blinded to the clinical presentation of the patients.

### Statistical Analysis

All statistical analyses were conducted using Prism software (version 6.0; GraphPad Software, La Jolla, CA, USA). The data are presented as the mean ± standard deviation or median with range. Continuous variables of the pediatric and adult groups were analyzed using the unpaired *t*-test or Mann-Whitney U test, and categorical variables were analyzed using the chi-squared test or Fisher's exact test. Statistical significance was set at *p* < 0.05. The detailed original data is available upon formal request for readers.

## Results

### Demographic Data and Clinical Characteristics

Table 1 summarizes the demographic and clinical characteristics of patients with MOG-EM enrolled in our study. A total of 53 patients, including 28 pediatric patients (age ≤18 years) and 25 adults (age >18 years), were admitted to our study. The average ages of onset in the pediatric and adult groups were 9.5 ± 0.66 years (range, 3–17 years) and 43.2 ± 2.32 years (range, 27–65 years), respectively. Twenty-two cases were male, and 31 were female; the male-to-female ratios of the pediatric and adult groups were 10:18 and 12:13, respectively (*p* = 0.4127).

**Table 1:** Comparison of the demographic and clinical characteristics of the pediatric and adult patients.

	**Pediatric group**	**Adult group**	***P*-valve**
Number	28	25	
Onset age in years, mean ± SD(range)	9.5 ± 0.66 (3–17)	43.2 ± 2.32 (27–65)	
Gender, M/F	10:18	12:13	0.4127
Prodromic infection	42.86% (12/28)	20% (5/25)	0.0751
**Initial phenotype**			
ADEM	17.86% (5/28)	8% (2/25)	0.5146
NMOSD	21.43% (6/28)	48% (12/25)	0.0414
BON	28.57% (8/28)	0% (0/25)	0.0119
UON	3.57% (1/28)	20% (5/25)	0.1470
EM/Myelitis	3.57% (1/28)	12% (3/25)	0.5230
Encephalitis/Menigoencephalitis	21.43% (6/28)	8% (2/25)	0.3276
MS	3.57% (1/28)	4% (1/25)	0.5220
**Onset symptoms**			
Fever	28.57% (8/28)	4% (1/25)	0.0442
Headache	32.14% (9/28)	24% (6/25)	0.5112
Visual impairment	39.29% (11/28)	64% (16/25)	0.0724
Encephalopathy	14.29% (4/28)	8% (2/25)	0.7743
Myelitis	21.43% (6/28)	32% (8/25)	0.3835
**Symptoms in full course**			
Fever	39.29% (11/28)	12% (3/25)	0.0245
Headache	50.00% (14/28)	24% (6/25)	0.0513
Visual impairment	57.14% (16/28)	72% (18/25)	0.2602
Encephalopathy	25.00% (7/28)	16% (4/25)	0.6403
Myelitis	28.57% (8/28)	40% (10/25)	0.3805

**Figure 1:**
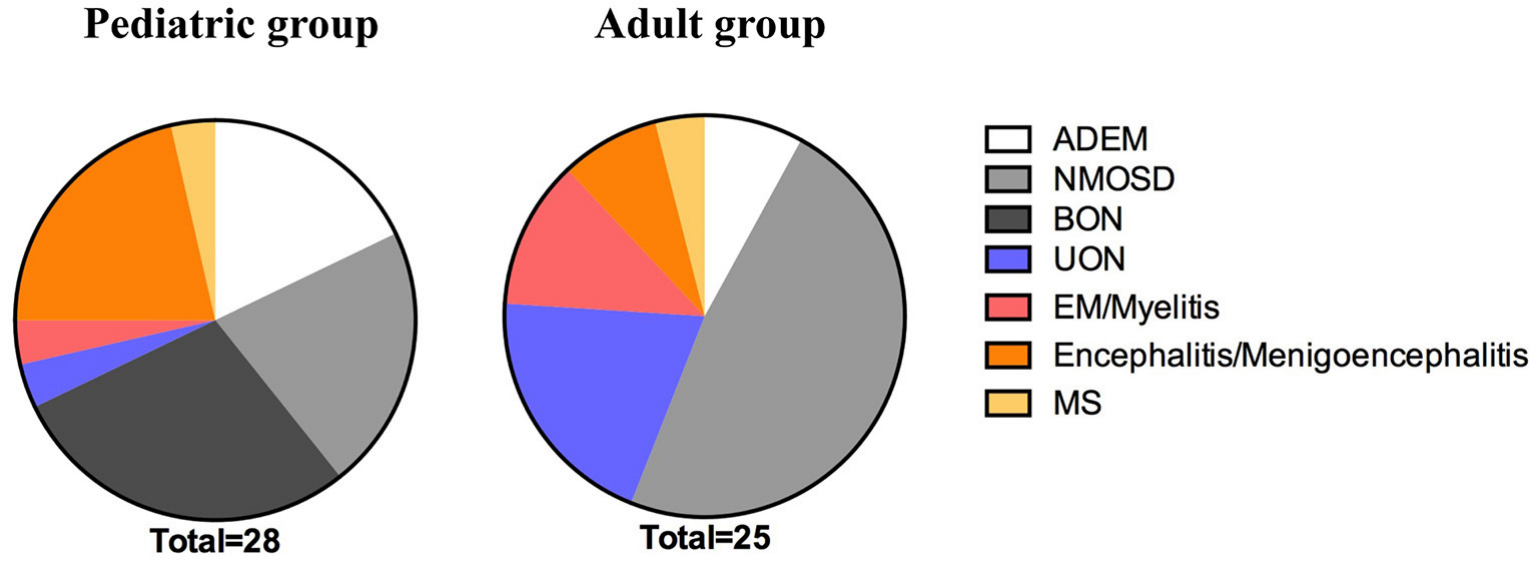
The initial phenotype of the pediatric and adult groups. The prevalence of BON was significantly higher in the pediatric patients than the adult patients during the initial attack (*p* = 0.0119). Fewer pediatric than adult cases met the criteria for NMOSD during the initial onset (*p* = 0.0414). ADEM, acute disseminated encephalomyelitis; NMOSD, neuromyelitis optica spectrum disease; BON, bilateral optic neuritis; UON, unilateral optic neuritis; EM, encephalomyelitis; MS, multiple sclerosis.

In the pediatric group, the most common initial phenotype was bilateral ON (BON) (28.57%, 8/28); it was more prevalent in the pediatric than in the adult group (0%, 0/25; *p* = 0.0119). However, almost half of the adult patients presented with NMOSD (48%, 12/25), which was more prevalent than in the pediatric patients (21.43%, 6/28; *p* = 0.0414). Of the pediatric cases, 7.86% (5/28) presented with ADEM, 21.43% (6/28) with encephalitis or menigoencephalitis, 3.57% (1/28) with unilateral ON (UON), 3.57% (1/28) with EM or myelitis, 3.57% (1/28) with MS; no significant difference was found between the pediatric and adult groups [8% (2/25) with ADEM, 8% (2/25) with encephalitis or meningoencephalitis, 20% (5/25) with UON, 12% (3/25) with EM or myelitis, and 4% (1/25) with MS] (Table 1 and Figure 1).

**Figure 2:**
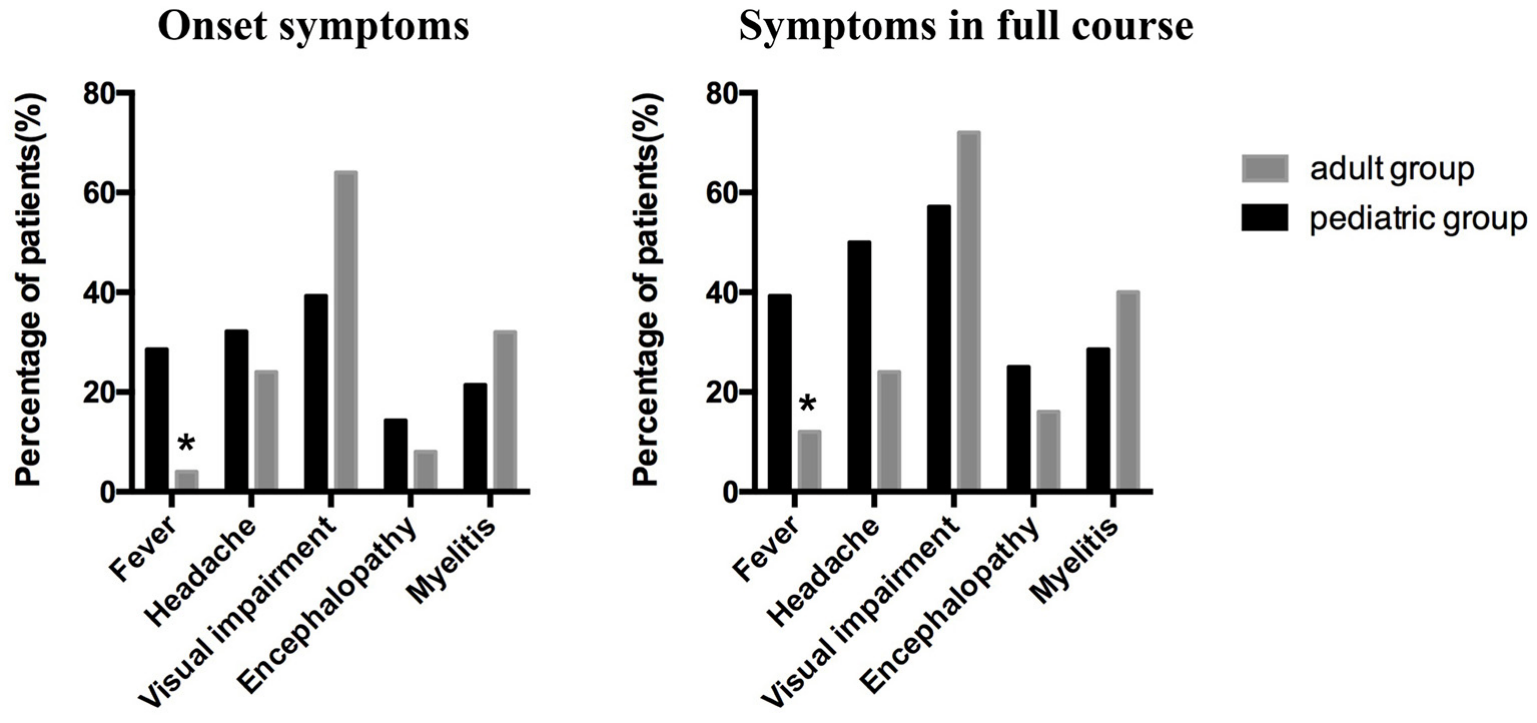
The clinical symptoms at the onset and throughout the full course of the disease in the pediatric and adult patients. Visual impairment, headache, and fever were the top three common symptoms in the pediatric group, whereas visual impairment, myelitis symptoms, and headache were the top three common symptoms in the adult group. More pediatric than adult patients suffered from fever at initial onset (*p* = 0.0442) and throughout the full course of the disease (*p* = 0.0245).

Visual impairment, headache, and fever were the top three common symptoms in the pediatric group, regardless of the initial attack or the full course [39.29% (11/28), 32.14% (9/28), and 28.57% (8/28), respectively, during the initial attack; 57.14% (16/28), 50.00% (14/28), and 39.29% (11/28), respectively, throughout the full course]. In the adult group, the top three common symptoms were visual impairment, myelitis symptoms (including urinary and fecal retention or incontinence, limb weakness and sensory dysfunction), and headache during the initial attack [64% (16/25), 32% (8/25), and 24% (6/25), respectively] and the full course [72% (18/25), 40% (10/25), and 24% (6/25), respectively]. However, fever was more frequent in the pediatric group than in the adult group during the initial attack (pediatric group, 28.57%; adult group, 4%; *p* = 0.0442) or the full course (pediatric group, 39.29%; adult group, 12%; *p* = 0.0245). Moreover, the headache proportion was slightly higher in the pediatric than in the adult group throughout the entire course (although not statistically significant, *p* = 0.0513); the proportions of headache in the two groups were similar at disease onset (*p* = 0.5112). There was no significant difference between the proportions of myelitis and encephalopathy in the pediatric and adult groups during the initial attack or the full disease course (Table 1 and Figure 2). We also observed that more pediatric patients (42.86%, 12/28) had respiratory tract infection before the initial onset than adult patients (20%, 5/25), although there was no statistical difference between the two groups (*p* = 0.0751) (Table 1).

### Laboratory Results

Twenty-five pediatric patients and 22 adult patients underwent CSF examination during the initial attack. Approximately half of the patients (51.06%, 24/47) had a normal WBC count of <5^*^106/L; 42.55% (20/47) had slightly elevated WBC counts of 6–100^*^106/L, and only 6.38% (3/47) had counts above 100^*^106/L. Most patients (84.78%, 39/46) had normal CSF protein concentrations of ≤450 mg/L, whereas only 1 of 46 (2.17%) patients had elevated protein concentrations of >1 g/L. There was no significant difference between the WBC counts and the CSF protein concentrations of the pediatric and adult groups. In our study, only two pediatric (8%, 2/25) and two adult (18.18%, 2/11) patients tested positive for the oligoclonal band (OCB) (Table 2).

**Table 2:** Comparison of the laboratory results of the pediatric and adult groups.

	**Pediatric group**	**Adult group**	***P*-valve**
**CSF**			
WBC ≤ 5*10^6^/L	48.00% (12/25)	54.55% (12/22)	0.6542
5*10^6^/L < WBC ≤ 100*10^6^/L	44.00% (11/25)	40.91% (9/22)	0.8307
**WBC** **≤** 100*10^**6**^**/L**			
WBC > 100*10^6^/L	8.00% (2/25)	4.55% (1/22)	0.9088
protein ≤ 450mg/l	84.00% (21/25)	85.71% (18/21)	0.8020
450mg/l < protein ≤ 1g/l	16.00% (4/25)	9.52% (2/21)	0.8335
protein > 1g/l	0% (0/25)	4.76% (1/21)	0.9297
anti-NMDAR antibody	8% (2/25)	9.09% (2/22)	0.8936
OCB positivity (%)	8.00% (2/25)	18.18% (2/11)	0.7491
Autoantibody positivity (%)	7.69% (2/26)	24% (6/25)	0.1094

Notably, 2 of 26 (7.69%) pediatric and 6 of 25 (24%) adult patients had serum-positive autoantibodies: one (3.85%) ANA positive and one (3.85%) TPO and TG antibody double-positive in the pediatric group; 2 (8%) ANA single positive, one (4%) TPO antibody single positive, one (4%) jo-1 antibody single positive, one (4%) ANA, Ro-52, SSA and SSB quadruple positive, and one (4%) Ro-52 and SSA double-positive in the adult group. Additionally, 2 of 25 (8%) pediatric patients and 2 of 22 (9.09%) adult patients had anti-N-methyl-D-aspartate receptor (anti-NMDAR) antibodies in their CSF; of these four cases, three had abnormal mental behavior and 2 had a speech disorder (Table 2).

### Magnetic Resonance Imaging Manifestations

**Figure 3:**
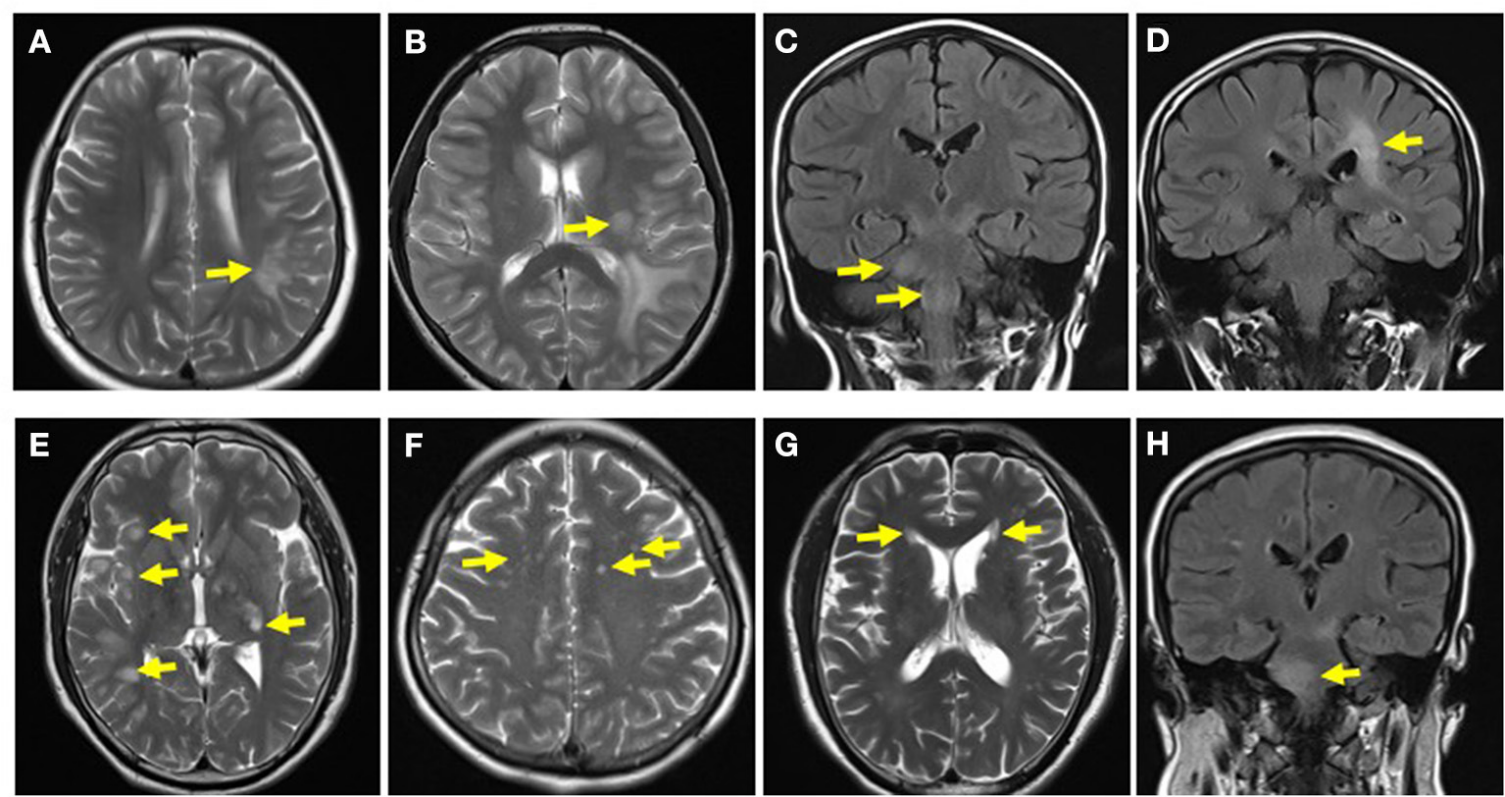
Brain MRI of pediatric and adult patients. **(A)** Subcortical white matter lesion in a pediatric patient. **(B)** Deep gray matter in a pediatric patient. **(C)** Infratentorial lesion in a pediatric patient. **(D)** Periventricular lesion in a pediatric patient. **(E)** Subcortical white matter and deep gray matter lesions in an adult patient. **(F)** Centrum semiovale lesions in an adult patient. **(G)** Periventricular lesions in an adult patient. **(H)** Infratentorial lesions in an adult patient.

Brain MRI was performed for 27/28 pediatric patients and 20/25 adult patients in our study. Abnormal brain lesions were detected in most adult cases (90%, 18/20); they were significantly more common in adult than in pediatric patients (48.15%, 13/27, *p* = 0.0028). Moreover, the subcortical white matter (pediatric group, 40.74%, 11/27; adult group, 45%, 9/20) and the periventricular (pediatric group, 25.93%, 7/27; adult group, 35%, 7/20), infratentorial (pediatric group, 18.52%, 5/27; adult group, 30%, 6/20), and deep gray matter (pediatric group, 25.93%, 7/27; adult group, 20%, 4/20) were the most common sites in all the cases, whereas cases involving the corpus callosum (pediatric group, 7.41%, 2/27; adult group, 5%, 1/20) were relatively rare. No significant difference was noted between the pediatric and adult groups. However, the proportion of patients with centrum semiovale involvement was higher in the adult group than in the pediatric group (35%, 7/20 vs. 3.70%, 1/27; *p* = 0.0151) (Table 3 and Figure 3).

**Table 3:** Comparison of the MRI manifestations of the pediatric and adult patients with MOG-EM.

	**Pediatric group**	**Adult group**	***P*-valve**
**Brain MRI**			
Abnormal	48.15% (13/27)	90% (18/20)	0.0028
Subcortical white matter	40.74% (11/27)	45% (9/20)	0.7703
Infratentorial lesion	18.52% (5/27)	30% (6/20)	0.5682
Centrum semiovale	3.70% (1/27)	35% (7/20)	0.0151
Periventricular	25.93% (7/27)	35% (7/20)	0.5012
Deep gray matter	25.93% (7/27)	20% (4/20)	0.8997
Corpus callosum	7.41% (2/27)	5% (1/20)	0.7875
**Optic nerve MRI**			
Abnormal	76.92% (20/26)	78.57% (11/14)	0.7811
Bilateral involvement	61.54% (16/26)	14.29% (2/14)	0.0042
Unilateral involvement	15.38% (4/26)	64.29% (9/14)	0.0052
**Spinal cord MRI**			
Abnormal	36.36% (8/22)	41.67% (5/12)	1.0000
Cervicothoracic segments	36.36% (8/22)	41.67% (5/12)	1.0000
Lumbosacral segments	4.55% (1/22)	0% (0/12)	1.0000
≥3 segments	18.18% (4/22)	25% (3/12)	0.6856

**Figure 4:**
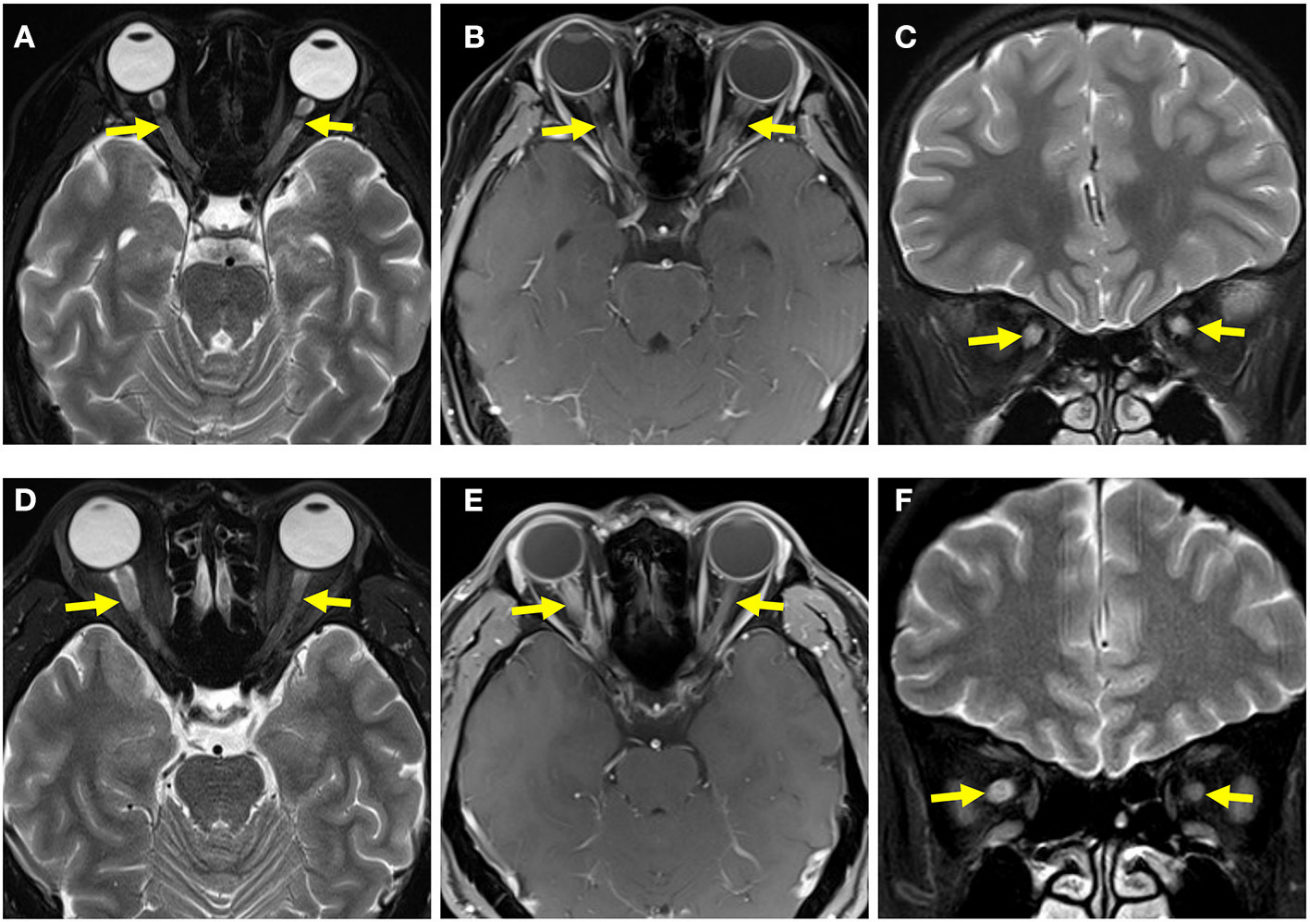
Optic nerve MRI of pediatric and adult patients. **(A)** T2-weighted MRI of the optic nerve of a pediatric patient showed bilateral optic-nerve thickening. **(B)** T1-weighted gadolinium MRI of the optic nerve in a pediatric patient showed bilateral optic-nerve enhancement. **(C)** Coronal image showed bilateral optic-nerve thickening. **(D)** T2-weighted MRI of the optic nerve of an adult patient showed that the right optic nerve was thicker than the left optic nerve. **(E)** T1-weighted gadolinium MRI of the optic nerve of an adult patient showed right optic-nerve enhancement. **(F)** A coronal image showed that the right optic nerve was thicker than the left optic nerve.

In the pediatric and adult groups, 76.92% (20/26) and 78.57% (11/14) of the cases presented with abnormal signals on optic nerve MRI, respectively. Notably, 61.54% (16/26) of the pediatric patients had bilateral optic nerve involvement; it was more prevalent in the pediatric than in the adult patients (14.29%, 2/14; *p* = 0.0042). In contrast, most adults had unilateral optic nerve involvement; it was significantly more prevalent in the adult than in the pediatric cases (64.29%, 9/14 vs. 15.38%, 4/26; *p* = 0.0052) (Table 3 and Figure 4).

**Figure 5:**
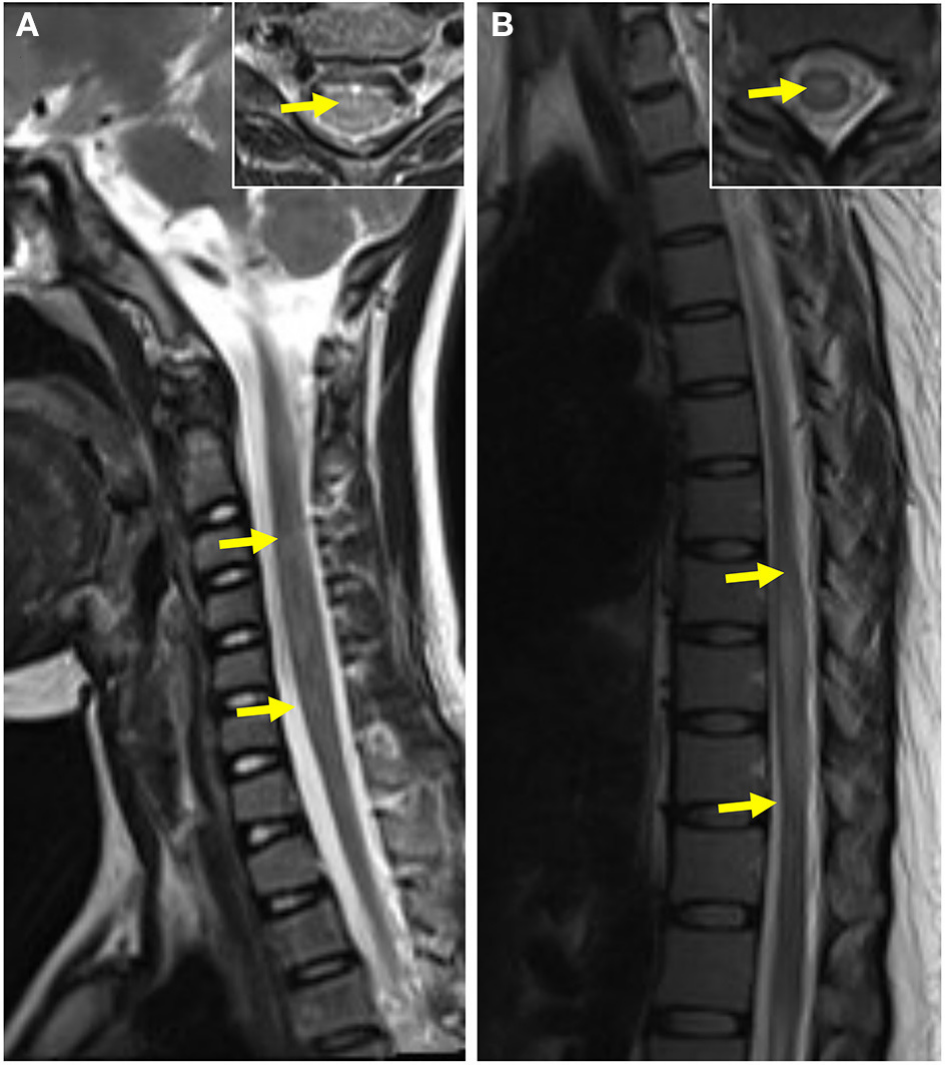
Spinal cord MRI of pediatric and adult patients. **(A)** T2-weighted MRI of the spinal cord of a pediatric patient showed abnormal patchy signals at the cervical segment The coronal image of lesion-involved slices was showed in the right box. **(B)** T2-weighted MRI of the spinal cord of an adult patient showed a striped hyperintense lesion at the thoracic segment. The coronal image of lesion-involved slices was showed in the right box.

Twenty-two of 28 pediatric cases and 12 of 25 adult cases had spinal cord MR scans in our study: 38.24% (13/34) cases showed abnormal signals for the spinal cord, and all of those lesions were distributed within the cervicothoracic segment; only one pediatric case had coexisting cervicothoracic and lumbosacral segment lesions. Furthermore, 20.59% (7/34) of the patients had spinal cord lesions distributed across three or more segments. There was no significant difference between the spinal cord lesions in the pediatric and adult groups (Table 3 and Figure 5).

### Treatment and Outcomes

Except for one pediatric patient and one adult patient who refused corticosteroid therapy during the initial attack, all the others received high-dose intravenous methylprednisolone followed by tapered oral steroid treatment. In the pediatric group, 20 of 28 (71.43%) patients received intravenous immunoglobulin, 11 of 28 (39.29%) patients who were initially suspected to have CNS bacterial infection accepted antibiotics, 15/28 (53.57%) cases who were initially suspected of having CNS viral infection received antiviral therapy, one (1/28, 3.57%) received azathioprine due to corticosteroid-induced high intraocular pressure, one (1/28, 3.57%) case was treated with azathioprine, and one (1/28, 3.57%) case was treated with mycophenolate mofetil (MMF) because of relapse. In the adult group, intravenous immunoglobulin was used only in 3 of 25 (12%) cases, antibiotics were used in 5/25 (20%) cases, and antiviral drugs were used in 5/25 (20%) cases during the initial attack. Methotrexate was used in one case (1/25, 4%), azathioprine was used in 6/25 (24%) cases, and MMF was used in 3/25 (12%) cases after oral steroids to prevent relapse. Moreover, 4/25 (16%) adult patients were treated with azathioprine, and one (1/25, 4%) was treated with interferon during relapse episodes. One (1/25, 4%) patient treated with azathioprine at the beginning was switched to MMF because of a decline in WBC, and one (1/25, 4%) treated with MMF at the beginning was switched to azathioprine because of low efficacy. One (1/28, 3.57%) patient in the pediatric group and one (1/25, 4%) in the adult group received rituximab during relapse episodes.

**Figure 6:**
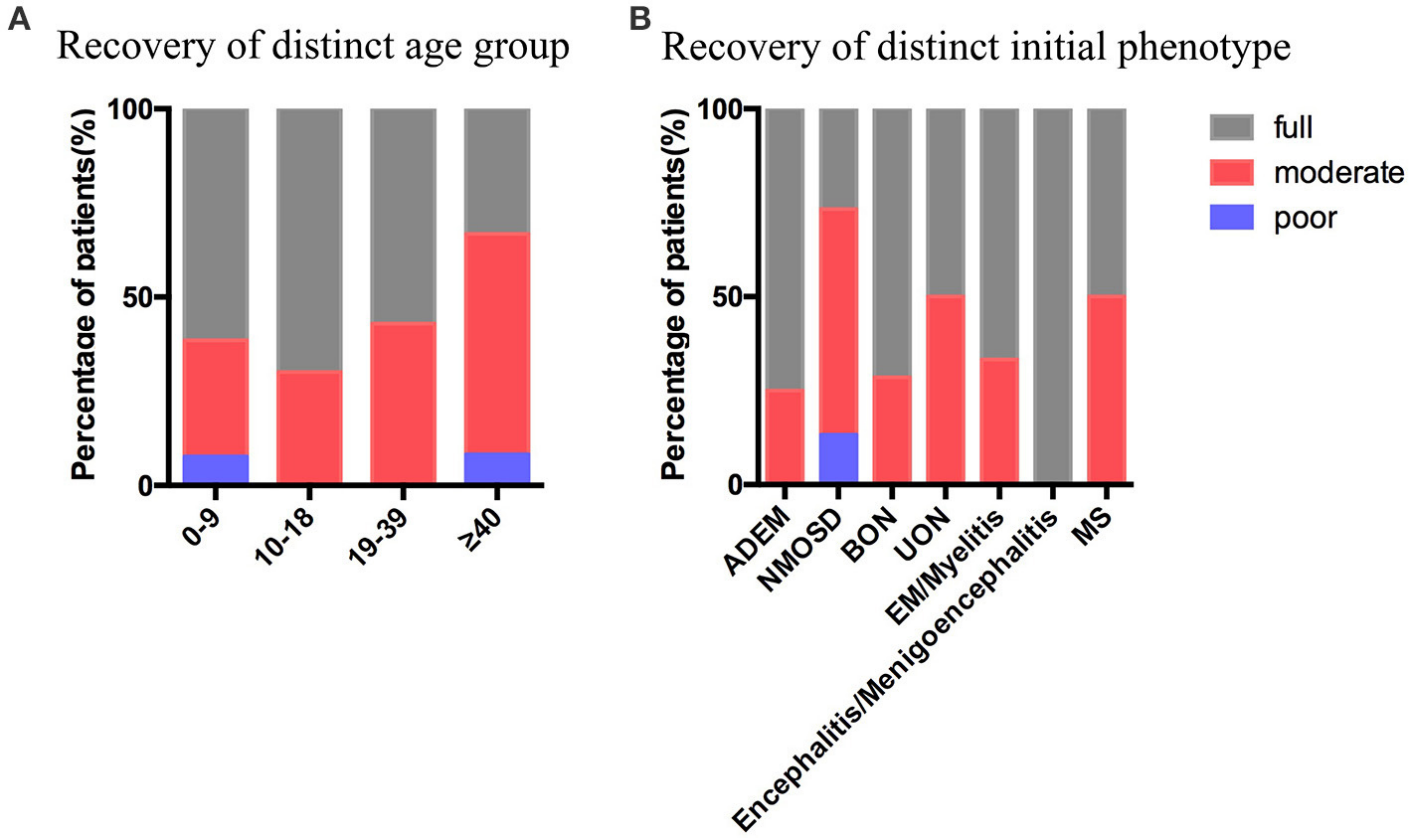
Recovery at the last follow-up. **(A)** Recovery at the last follow-up stratified by the age groups. **(B)** Recovery at the last follow-up stratified by the initial phenotype. ADEM, acute disseminated encephalomyelitis; NMOSD, neuromyelitis optica spectrum disease; BON, bilateral optic neuritis; UON, unilateral optic neuritis; EM, encephalomyelitis; MS, multiple sclerosis. Full recovery was defined as EDSS=0, moderate recovery was defined as 0 < EDSS ≤ 2, poor recovery was defined as EDSS ≥ 3.

In our study, the average follow-up durations in the pediatric and adult groups were 17 (range, 3–58) and 20 (range, 2–41) months, respectively. At the last follow-up, 5 of 23 (21.74%) pediatric patients and 5 of 19 (26.23%) adult patients had multiphasic course, 18 of 23 (78.26%) pediatric cases and 14 of 19 (73.68%) adult cases had monophasic course, both with *p*-value of 0.9862 (Table 4). In the pediatric group, the initial phenotypes of the five recurrent cases were BON (2), UON (1), NMOSD (1), and MS (1); in the adult group, four of the five recurrent cases presented with NMOSD, and the other one with MS. The average EDSS scores of the pediatric (median 3.0, range 0–9.5) and adult (median 3.0, range 0–9.5) groups were similar during the initial attack (*p* = 0.4937); however, the EDSS score was suggested to be slightly higher in the adult group (median 1.0, range 0–3) than in the pediatric group (median 0.0, range 0–3) at the last follow-up, though not statistical significant (*p* = 0.0752) (Table 4). In our study, all the patients were alive at the last follow-up, and the proportion of full recovery cases in the patients aged 0–9 years (61.54%) and 10–18 years (70%) was higher than that in the patients aged 19–39 years (57.140%) and >40 years (33.33%). Patients with encephalitis/meningoencephalitis (100%) and ADEM (75%) during the initial attack were more likely to have full recovery than those with NMOSD (26.67%), UON (50%), MS (50%), EM/myelitis (66.67%), and BON (71.43%) (Figure 6).

**Table 4:** Disease course and EDSS scores of the pediatric and adult patients with MOG-EM.

	**Pediatric group**	**Adult group**	***P*-valve**
Follow-up duration in month, median (range)	17 (3–58)	20 (2–41)	0.7541
Disease course (%)	21.74% (5/23)	26.32% (5/19)	0.7289
Monophasic	78.26% (18/23)	73.68% (14/19)	0.9862
Multiphasic	21.74% (5/23)	26.32% (5/19)	0.9862
EDSS at initial presentation, median (range)	3.0 (0–9.5)	3.0 (0–9.5)	0.4937
EDSS at last follow-up, median (range)	0.0 (0–3)	1.0 (0–3)	0.0752

## Discussion

Over the past few years, immunoglobulin G serum antibodies against MOG have been thought to be involved in IIDDs, including ADEM, ON, NMOSD, MS, and CIS (4–6). Since MOG antibody-related demyelination disease presents with typical clinical characteristics and has treatment outcomes distinct from other IIDDs, it is now considered an independent disease called MOG-EM (9). Even though MOG-EM is now well-detected, research on the differences between pediatric and adult patients is relatively rare and limited. Here, we collected cases with MOG-EM from the pediatric, neurology, and ophthalmology departments in our hospital and compared the differences between the clinical manifestations, radiologic presentations, and prognoses of the pediatric and adult patients with MOG-EM.

In this study, we collected 28 pediatric cases and 25 adult cases with MOG-EM, and found no gender difference between the pediatric and adult groups, which is similar to previous studies (14, 15). BON is the most frequent initial phenotype in the pediatric group followed by NMOSD and encephalitis/meningoencephalitis, whereas NMOSD is the most common initial phenotype, followed by UON and EM/myelitis in the adult group. However, several previous studies have demonstrated that ADEM is the most common phenotype in pediatric cases (11, 16, 17). In our opinion, collecting cases from the Ophthalmology Department may raise the proportion of patients with ON; some cases who only suffer from visual impairment may be admitted to the Ophthalmology Department rather than the Pediatric department, which probably accounts for the underestimation of the proportion of ON by previous studies. On the other hand, the diagnostic criteria for ADEM need to be more carefully considered, they should include the manifestations of encephalopathy, which includes the disturbance of consciousness and abnormal behavior. Although some patients have typical ADEM like brain MRI lesions, they may not present with encephalopathy symptoms, but rather present with encephalitis or meningitis symptoms, including headache, seizure, and fever. Visual impairment was the most common symptom in both pediatric and adult groups, which is consistent with our finding that BON and NMOSD were the most frequent phenotypes in the pediatric group, and NMOSD and UON were the most frequent phenotypes in the adult group. However, our data showed a higher prevalence of NMOSD than other's research at initial phenotype in adult group. As a recent study by analyzing the clinical profile of NMOSD in Australia and New Zealand found that prevalence of NMOSD in adult varies between 10 and 25% (18). Future cohort study with bigger sample size is still warranted for our research. We further noted that fever was more common in the pediatric group than in the adult group, which is similar to the findings of Mao et al. They suggested that the proportion of patients with fever was higher in children aged ≤9 years than in those older than 9 years (14).

Similar to previous studies (9, 19, 20), almost half of the patients in our cohort had normal WBC counts in the CSF, half had slightly elevated WBC levels, and the majority had normal CSF protein concentrations. This suggests that MOG-EM should be differentiated from viral encephalitis. Only a few patients had positive CSF OCB in MOG-EM, which implies that MOG-EM should be carefully diagnosed when the patient has positive CSF OCB. Similar to the study by Hou et al. (21), we found four cases with MOG-EM coexisting with the presence of the anti-NMDAR antibody; three of them had abnormal mental behavior and two had speech disorder, indicating that patients with anti-NMDAR encephalitis, especially those with abnormal mental behavior or a speech disorder, should be tested for the MOG antibody.

Multiple patchy lesions in the subcortical white matter and the periventricular, infratentorial, and deep gray matter were the most common brain lesions in both pediatric and adult patients in our study, while those in the corpus callosum were relatively rare. This was emphasized in previous studies, and it is consistent with ADEM lesions (22, 23); however, only 17.86% of pediatric cases and 8% of adult cases in our study presented with ADEM during the initial attack, implying that the clinical presentations of MOG-EM are not congruent with its image manifestations. Most patients in the pediatric and adult groups had abnormal optic nerve signals in the present study, which partly explains the high proportion of patients with visual impairment. In contrast with reports by previous studies (24, 25) that most patients with MOG-EM had spinal cord lesions located in the lumbosacral segments, our study found that cervicothoracic segment lesions were the most common in pediatric and adult patients, which is consistent with the findings of a Chinese study by Chen et al. (11). These differences may be due to racial differences and the limited sample size.

In this study, most cases in the pediatric group were admitted corticosteroid and intravenous immunoglobulin therapy during the initial attack, and only four patients received immunosuppression therapy during the relapse episodes. However, in the adult group, only 12% of the patients were admitted intravenous immunoglobulin; 40% received immunosuppressive therapy after corticosteroids during the initial attack. This phenomenon implies that pediatric patients are more likely to receive intravenous immunoglobulin therapy because of their immunomodulatory effects and the lack of immunosuppression. In our study, over 70% of the patients in this study had monophasic course during follow-up, which was in line with previous research (26). In addition, the patients presenting with ON and NMOSD were more likely to relapse, which was consistent with previous studies (27–29). In our cohort, we found that the median EDSS score in the pediatric group (median 0.0, range 0–3) was suggested to be lower than in the adult group (median 1.0, range 0–3) at the last follow-up (*p* = 0.0752), suggesting that pediatric patients tend to have better outcomes than adult patients although both have good prognoses. Additionally, our data suggest that pediatric and adolescent patients are likely to have a better recovery than adult patients at the last follow-up, and patients presenting with encephalitis/meningoencephalitis and ADEM during the initial attack have a higher proportion of full recovery cases than others, especially those with NMOSD, UON and MS. A UK study also emphasized that younger patients were more likely to fully recover than older adults, and patients with ADEM more frequently had full recovery (30).

Our study has several limitations. First, it is relatively a small-sample research; MOG-EM is a new disease that has only been defined in recent years, and the number of patients diagnosed is limited. Second, this was a retrospective study; therefore, selection bias was difficult to avoid, and clinical data collection was limited. Future prospective studies with large sample sizes and long follow-up durations are required to investigate the effect of the age of onset on the clinical and radiological manifestations and the prognosis of MOG-EM. Biological research is also warranted to explain the differences between children and adults.

Even though there are common clinical and radiologic presentations in pediatric and adult cases of MOG-EM, there are also differences. Visual impairment was the dominant symptom in both pediatric and adult patients, whereas fever was more frequent in pediatric patients. In the pediatric group, BON and bilateral optic nerve involvement were more common, while NMOSD and unilateral optic nerve involvement were more frequent in adult patients. Though none BON was found in adult patients in our data, it's should be noted that BON is not rare in other study (31, 32), differences in sample size and potential bias may contribute to above different results. Multiple patchy intracranial lesions in the subcortical white matter and periventricular, infratentorial, and deep gray matter were prevalent in both age groups. The prognosis is good in all patients after treatment, but younger patients and patients presenting with encephalitis/meningoencephalitis and ADEM tend to have better recovery than the others.

## Data Availability Statement

The raw data supporting the conclusions of this article will be made available by the authors, without undue reservation.

## Ethics Statement

The studies involving human participants were reviewed and approved by Ethics Committee of the Second Xiangya Hospital of Central South University. Written informed consent to participate in this study was provided by the participants' legal guardian/next of kin.

## Author Contributions

JXu and LiqL contributed to the conception of this article. LiqL, LinL, JXi, YX, XL, JL, YL, HL, and DM provided the clinical information. JXu, LZ, LT, and PH analyzed the data. JXu wrote the draft of the manuscript. Final integration and editing were done by LiqL. All authors contributed to the article and approved the submitted version.

## Conflict of Interest

The authors declare that the research was conducted in the absence of any commercial or financial relationships that could be construed as a potential conflict of interest.
